# Evaluation of the diagnostic value of circulating tumor cells with CytoSorter^®^ CTC capture system in patients with breast cancer

**DOI:** 10.1002/cam4.2825

**Published:** 2020-01-06

**Authors:** Lidan Jin, Wenhe Zhao, Jun Zhang, Wenjun Chen, Tan Xie, Linbo Wang, Wanhung Fan, Shuduo Xie, Jianguo Shen, Heming Zheng, Wenxian Hu, Qun Wei, Minjun Dong, Qinchun Wang, Jun Shen, Yongcheng Liu

**Affiliations:** ^1^ Department of Surgical Oncology Sir Run Run Shaw Hospital Affiliated to Zhejiang University College of Medicine Hangzhou China; ^2^ Department of Clinical Laboratory Sir Run Run Shaw Hospital Affiliated to Zhejiang University College of Medicine Hangzhou China; ^3^ Department of Nursing Sir Run Run Shaw Hospital Affiliated to Zhejiang University College of Medicine Hangzhou China; ^4^ Hangzhou Watson Biotech Hangzhou China

**Keywords:** breast cancer, cancer staging, circulating tumor cells, early diagnosis, screening

## Abstract

**Purpose:**

In this study, we aimed to investigate the viability of utilizing CytoSorter^®^ system to detect circulating tumor cells (CTCs) and to evaluate the diagnostic value of CTCs in breast cancer (BC).

**Methods:**

A total of 366 females patients suspected of having BC and 30 healthy female volunteers were enrolled in this study. CTCs were enriched by CytoSorter^®^, a microfluidic‐based CTCs capturing platform. CTC detection was performed before operation or biopsy. Based on the biopsy results, patients were divided into two groups, namely patients with BC and patients with benign breast diseases (BBD). Patients with BBD and healthy volunteers were serving as controls. The correlation between CTC enumeration and patients' clinicopathological characteristics was evaluated. The receiver operating characteristic (ROC) curve was plotted to assess the diagnostic potency of CytoSorter^®^ system in BC.

**Results:**

Based on the biopsy results, 130 BC patients at different cancer stages and 236 patients with BBD were enrolled in the study. Seven subjects were dropped out from the study. CTCs were detected in 109 of 128 BC patients, in one of 29 healthy volunteers, and in 37 of 232 patients with BBD. Maximum CTC counts detected in BC patients, healthy volunteers, and patients with BBD were 8, 1, and 4, respectively. Statistical analysis showed CTCs could be used to distinguish BC patients from healthy volunteers and patients with BBD (*P* < .0001). Circulating tumor cells were statistically associated with patients' cancer stage (*P* = .0126), tumor size (tumor node metastasis [TNM] T stage, *P* = .0253), cancer type (invasive vs noninvasive, *P* = .0141), and lymph node metastasis (*P* = .0436). More CTCs were found in patients at advanced cancer stage or TNM T stage and in patients with invasive tumor or lymph node metastasis. Furthermore, CTC detection rates in BC patients at Tis and T1‐4 stages were 50%, 81.67%, 91.07%, 100%, and 100%, respectively. When the CTC cut‐off value was set to 2, the ROC curve gave an area under the curve (AUC) of 0.86 with a specificity and sensitivity of 95.4% and 76.56%, respectively. Taken together, CTCs could be used as a diagnostic aid in assistance of cancer screening and staging.

**Conclusion:**

Circulating tumor cells were successfully isolated in BC patients using CytoSorter^®^ system. CTCs can be used to differentiate BC patients from the patients with BBD or healthy volunteers, and as a diagnostic aid for early cancer diagnosis and cancer staging.

## Graphical abstract

Circulating tumor cells were successfully isolated in breast cancer (BC) patients using CytoSorter^®^ system. Circulating tumor cells can be used to differentiate BC patients from the patients with benign breast diseases or healthy volunteers, and as a diagnostic aid for early cancer diagnosis and cancer staging.

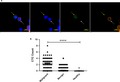

## 1 INTRODUCTION

Breast cancer (BC) is one of the most common malignant tumors. In 2018, there were more than 2.1 millions of newly diagnosed cases of BC and it has caused over 630 000 deaths throughout the world.^1^ Breast cancer is the most common cause of tumor‐related deaths among women in more than 100 countries.^2^ Although the incidence of BC is usually higher in Caucasian women, BC is still the most common tumor in female in China. In 2015, it was estimated that 304 000 BC cases were newly diagnosed among women and approximately 69 900 women died of BC in China.^3^ Mortality rates of BC in developed countries are decreasing, whereas incidence and mortality rates of BC in developing countries such as China are still increasing.^1^ Increased survival in BC patients is mainly due to the improvement of the screening methods, early diagnosis, and breakthroughs in treatments.

The conventional methodologies for diagnosis of BC include imaging methods, breast biopsy, and blood‐based assay.^4^ To increase diagnostic accuracy and eliminate false‐negative results, clinical breast examination, breast imaging, biopsy, and blood test are usually performed simultaneously.^5^ Imaging methods in BC include ultrasound, mammography, magnetic resonance imaging (MRI), and molecular breast imaging. Improvements in imaging techniques have led to increased sensitivity, although these techniques are still not so sensitive to detect the tumor at a very early stage.^6^ Biopsy is the gold standard for diagnosing BC although it cannot be performed frequently. Blood samples can be easily obtained, but serum BC‐specific biomarkers, such as cancer antigen 15‐3 (CA 15‐3) and carcinoembryonic antigen (CEA), have low sensitivity and specificity, and thus are not useful in the early detection of BC.^7^ American Society of Clinical Oncology recommends the use of CEA and CA 15‐3 only in metastatic BC (MBC).^8^ To improve the survival of BC patients, it is necessary to find a reliable biomarker allowing better cancer screening and early diagnosis.

Circulating tumor cells (CTCs) are tumor cells that have shed from the primary tumor or metastatic tumors and entered the peripheral blood circulation. Studies have shown that CTCs play an important role in tumor metastasis and have prognostic values in BC patients.^9^, ^10^, ^11^ The 7th edition of the American Joint Committee on Cancer (AJCC) Staging Manual for BC has introduced a cM0(i+) stage for patients without clinical or radiographic evidence of distant metastases but with tumors cells detected in the bone marrow (ie, disseminated tumor cells), in blood (ie, CTCs) or in distant nonregional lymph nodes. In the 8th edition of the AJCC cancer guidelines, it is written that CTCs can be used as a prognostic factor in BC to predict patients’ survival outcome, that is, progression‐free survival (PFS) and overall survival (OS). Patients with MBC usually have more CTCs and BC patients with more CTCs usually have shorter PFS and OS.^9^, ^10^, ^11^ Moreover, CTCs can be used as a monitoring tool to evaluate patients' response to the treatment and to see whether tumor recurrence occurs.^12^, ^13^

Circulating tumor cells are rare in the blood and thus many techniques have been developed to enrich CTCs from the blood based on the unique physical or biological properties of CTCs. As the first and only US Food and Drug Administration approved CTCs detection system, CellSearch^®^ utilizes an immunomagnetic method to capture epithelial CTCs. Studies with CellSearch^®^ system have shown CTCs detection rate in BC patients was less than 40%.^14^, ^15^, ^16^ Enrichment of CTCs using microfluidics methods, such as the herringbone‐Chip (HB‐Chip), showed a better detection sensitivity.^17^ The CytoSorter^®^ (Hangzhou Watson Biotech), a microfluidic‐based immune capturing platform, uses a HB‐Chip called CytoChipNano to enrich CTCs, and the preliminary data showed CTCs detection rate was more than 70% in BC with CytoSorter^®^. Therefore, we decided to use CytoSorter^®^ CTCs detection system in this study and to evaluate its diagnostic potency in BC. The clinical application of CytoSorter^®^ has been reported in pancreatic cancer and head and neck cancers.^18^, ^19^ CytoSorter^®^ technology employs the positive selection utilizing a streptavidin nanoarray on CytoChipNano, which can be coated with any desired biotin‐labeled capture antibody (Ab), and immunofluorescence staining, to capture and identify CTCs. Capture and identification antibodies for CTCs used in this study are anti‐epithelial cell adhesion molecules (EpCAM) and anti‐pan‐cytokeratin (PanCK), respectively.

A total of 366 patients suspected of having BC and 30 healthy volunteers were enrolled in this study. Based on the biopsy results, the patients were then divided into BC patients and patients with benign breast diseases (BBD). Circulating tumor cell detection was performed before biopsy or operation and its correlation with patients' clinicopathological findings would be analyzed. The aims of this study were as follows: (a) to assess the viability of CTCs detection in BC using CytoSorter^®^ system; (b) to correlate CTCs to BC patients' clinicopathological findings; (c) to evaluate CTCs as a marker for early diagnosis and cancer staging of BC; and (d) to evaluate the diagnostic potency of CytoSorter^®^ system in BC.

## 2 MATERIALS AND METHODS

### 2.1 Ethics

The study followed the principles established in the Declaration of Helsinki and was approved by the ethics committee of Zhejiang University Medical College Affiliated Sir Run Run Shaw Hospital. The written consent for participation in this research and publication of their case details was obtained from each patient and healthy volunteer.

### 2.2 Cell Lines

The human breast adenocarcinoma cell line, SK‐BR‐3 (TCHu225), was obtained from the Cell Bank of Chinese Academy of Sciences. SK‐BR‐3 cells were maintained in Dulbecco's Modified Eagle's Medium (DMEM) (Gibco, Thermo Fisher Scientific), supplemented with 10% fetal bovine serum (Gibco, Thermo Fisher Scientific) in the presence of penicillin and streptomycin. SK‐BR‐3 cell was cultured in 37°C incubators with 5% CO_2_ saturation.

### 2.3 Patients

In total, 366 female patients suspected of having BC, and 30 healthy females were enrolled in this study between December 2017 and November 2018. Inclusion criteria were as follows: (a) female patients aged 18‐75 years; (b) patients suspected of having BC, in whom breast masses were found by palpation, ultrasound and/or mammography, and planning to have puncture biopsy or operation; (c) patients had negative history of malignancy, and were treatment‐naive before enrollment; (d) healthy individuals had no medical history of any malignant disease and no findings in breast by palpation, ultrasound and/or mammography; (e) patients had signed up the consent forms and were compliant to the examinations and blood sample collection. Exclusion criteria were as follows: (a) patients were pregnant or breast‐feeding; (b) patients were currently undergoing or had prior cancer treatment; (c) patients had other malignant tumors or diseases within 5 years prior to enrollment; (d) patients had other conditions which investigators thought was not suitable for the study. Subjects of the following descriptions were rejected from the study: (a) no clear histopathological diagnosis of tissue biopsy or unknown tumor node metastasis (TNM) staging; (b) white blood cells (WBC) count was greater than 12 × 10^9^/L or less than 2 × 10^9^/L; (c) Eastern Cooperative Oncology Group score >2; (d) blood sample collection and preservation did not follow standard procedure; (e) blood samples were not processed within 6 hours after collection; (f) collected blood sample was less than 4 mL; (g) blood clotting in the blood sample; (h) any abnormality during sample processing; (i) hemolysis in the blood sample.

### 2.4 Blood collection and preparation

The first 2 mL of collected peripheral blood was discarded to avoid potential skin cell contamination from venipuncture. Collected blood (5‐10 mL) was stored in a heparin tube (BD). Blood has maximum preservation time of 6 hours at room temperature. CTCs were enriched by CytoSorter^®^ system. Blood preprocessing procedure was described as in the manufacture protocol. In brief, 4 mL of blood sample was diluted at 1:1 ratio with 1X PBS to final volume of 8 mL, and then transferred equally into 2 separate Leucosep^®^ tubes containing 2 mL of Histopaque^®^‐1077 (Sigma‐Aldrich) density gradient media. After density gradient centrifugation, peripheral blood mononuclear cells (PBMCs) layer was isolated and washed twice with washing medium (WM, 5% FBS DMEM). Final cell pellet was re‐suspended in 190 μL of WM and ready for further use.

### 2.5 CTC detection

Circulating tumor cells were enriched by CytoSorter^®^ epithelial cells detection kit. Circulating tumor cell detection procedure was described as in the previous study.^19^ In brief, the CytoChipNano was first coated with EpCAM capture Ab before placing onto CytoSorter^®^ system. Aforementioned PBMCs sample solution was then transferred into SCx spiral sample tube. Once the CTCs enrichment was finished, the CytoChipNano was removed from CytoSorter^®^, followed by immunofluorescence staining of PanCK‐fluorescein isothiocyanate (FITC), CD45‐PE, and 4′,6‐diamidino‐2‐phenylindole (DAPI). Olympus scanning microscope (Olympas BX61) and CytoView™ software were used to scan CytoChipNano for potential CTCs, and then Nikon microscope (Nikon ECLIPSE Ti) was used to confirm CTCs staining and localization. CTCs were defined as PanCK‐FITC^+^, CD45‐PE^‐^, and DAPI^+^ cells.

### 2.6 Statistical analysis

All statistical analyses were performed using Prism 6.0 (GraphpadA) and SPSS 2.0 (IBM). A paired or unpaired Student's *t* test was used for continuous variables, as appropriate. The Chi‐squared test and Fisher's exact test were adopted for the comparison of categorical parameters. One‐way ANOVA was performed to calculate the differences among multiple groups. The receiver operating characteristic (ROC) curve was plotted to evaluate the sensitivity, specificity, and area under the curve (AUC) value of the system. Circulating tumor cell cut‐off value was determined by Youden index (sensitivity + specificity − 1). A two‐sided *P* value less than .05 was considered statistically significant.

## 3 RESULTS

### 3.1 Identification of CTCs in BC patients

**Figure 1:**
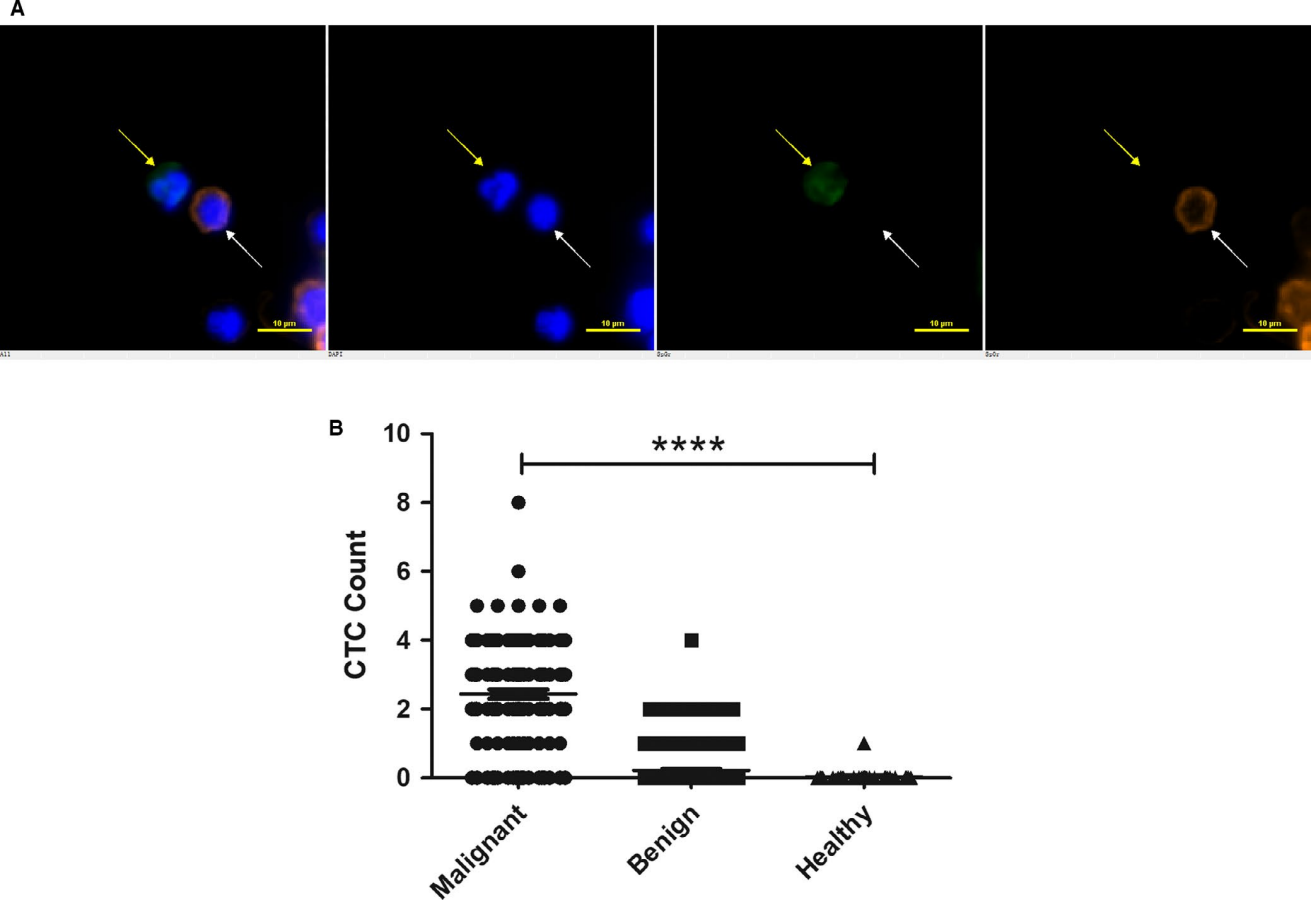
Circulating tumor cells (CTCs) detection in BC patients. A, Immunofluorescent staining of CTCs in breast cancer (BC) patients. CTCs are identified as DAPI (blue) positive, PanCK (FITC, green) positive, and CD45 (PE, orange) negative cells. CTC is indicated by the yellow arrow, whereas white blood cells are marked by white arrow. Scale bar represents 10 μm, immunofluorescent staining, X 20 (B) CTCs enumeration is able to differentiate BC patients from the healthy volunteers and patients with benign breast diseases (*P* < .0001). ****P < .0001

SK‐BR‐3 cell line was used as a quality control to evaluate the efficiency of CytoSorter^®^ system, and a capture rate of 92% was obtained with CytoSorter^®^ epithelial cells detection kit (data not shown). Circulating tumor cells are defined as PanCK‐positive, CD45‐negative, and DAPI‐positive cells as shown in Figure 1A. Based on the biopsy results, 366 patients suspected of having BC were divided into one group of 130 BC patients and another group of 236 patients with BBD, including breast fibroadenomas, breast adenosis, mammary duct ectasia, breast cysts, and fat necrosis of the breast. Seven subjects were later excluded from the study (one patient without pathological outcome, one patient with WBC count more than 12 × 10^9^, and five patients withdrew their consents). According to the TNM staging system of AJCC (7th edition), the number of enrolled BC patients at cancer stage 0, I, II, III, IV were, respectively, 6, 46, 59, 16, and 1 as shown in Table 1. A summary of the statistical results of CTCs based on patients' clinicopathological characteristics, including cancer type, cancer stage, and TNM classification, is listed in Table 1. Circulating tumor cells were detected in 109 of 128 BC patients and the average CTC count per 4 mL of blood is 2.44. Circulating tumor cells were detected in one healthy individual and 37 patients with BBD, with average CTC counts of 0.03 and 0.22, respectively. The range of CTC counts in BC patients, patients with BBD, and healthy volunteers are, respectively, 0‐8, 0‐4, and 0‐1. ANOVA result indicates that CTC enumeration is able to differentiate BC patients from healthy volunteers and patients with BBD (*P* < .0001) as shown in Figure 1B.

**Table 1:** Statistical analysis of CTCs among different groups of patients based on clinicopathological features

Subjects	N	CTC Positive	CTC detection rate (%)	Average CTC count (range)	*P* value
Healthy	29	1	3.45	0.03 (0‐1)	**<.0001**
Benign	232	37	15.95	0.22 (0‐4)	
Malignant	128	109	85.16	2.44 (0‐8)	
Cancer type					
Noninvasive	6	3	50	1.00 (0‐2)	**.0141**
Invasive	122	106	86.89	2.51 (0‐8)	
Cancer stage					
0	6	3	50	1.00 (0‐2)	**.0126**
I	46	37	80.43	2.17 (0‐4)	
II	59	54	91.53	2.59 (0‐6)	
III	16	14	87.5	3.25 (0‐8)	
IV	1	1	100	1	
TNM stage					
Tumor stage					
Tis	6	3	50	1.00 (0‐2)	**.0253**
T1	60	49	81.67	2.35 (0‐8)	
T2	57	52	91.23	2.58 (0‐5)	
T3	3	3	100	4.00 (4‐5)	
T4	2	2	100	3.00 (2‐4)	
Nodal stage					
N0	78	64	82.05	2.21 (0‐5)	.1871
N1	34	31	91.18	2.71 (0‐6)	
N2	8	6	75	2.5 (0‐4)	
N3	8	8	100	3.5 (1‐4)	
Nodal metastasis					
No	78	64	82.05	2.21 (0‐5)	**.0436**
Yes	50	45	90	2.8 (0‐6)	
Molecular subtype^a^					
Luminal A	69	57	82.6	2.51 (0‐6)	.675
Luminal B	17	12	70.59	2.06 (0‐4)	
HER2‐enriched	14	14	100	2.5 (1‐5)	
Triple negative	21	19	90.48	2.38 (0‐8)	

Bold indicates statistically significance values. Abbreviations: CTC, circulating tumor cell; N, number; TNM, tumor node metastasis classification.

a Due to the missing information, seven patients were removed from this analysis.

### 3.2 Correlation of CTC enumeration with patients’ clinicopathological characteristics

**Figure 2:**
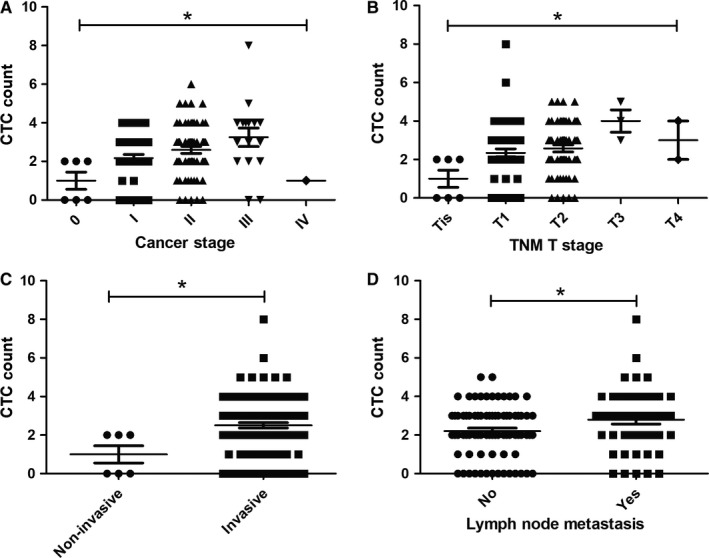
Circulating tumor cells (CTCs) were correlated with patients' cancer stage, tumor size, cancer type, and lymph node metastasis. More CTCs were found in patients at advanced cancer stage and TNM T stage as shown in (A) and (B) (*P* = .0126 and .0253, respectively). More CTCs were found in patients with invasive tumor and lymph node metastasis as shown in (C) and (D) (*P* = .0175 and .0436, respectively). .01 < *P < .05

In order to assess whether CTCs were associated with patients' clinicopathological characteristic, statistical analysis was performed among different groups of patients based on their age, TNM stages, cancer type, and cancer stages. As for patients with BBD, no statistical significance was found among patients with different BBD (data not shown). As for BC patients, analysis results showed CTCs were not related with patients' age (data not shown) and molecular subtype, but with cancer stage, tumor size, cancer type, and lymph node metastasis as shown in Table 1 and Figure 2. For only one stage IV BC patient was present in this study, she was excluded from the analysis. More CTCs were found in patients at advanced cancer stage (*P* = .0126). Average CTC count of patients at cancer stage from 0 to III were 1, 2.17, 2.59, and 3.25, respectively. Circulating tumor cell enumeration was correlated with tumor size as well. As shown in Table 1, patients with bigger tumor (at advanced TNM T stage) had more CTCs detected and a higher CTC detection rate (*P* = .0253). Patients with invasive tumor or lymph node metastasis had more CTCs as well compared with patients with noninvasive tumor or without lymph node metastasis (*P* = .0141 and .0436, respectively). However, CTCs were not correlated with patients' nodal stage (*P* = .1871).

### 3.3 Evaluation of diagnostic potency of CytoSorter^®^ system in BC patients

**Figure 3:**
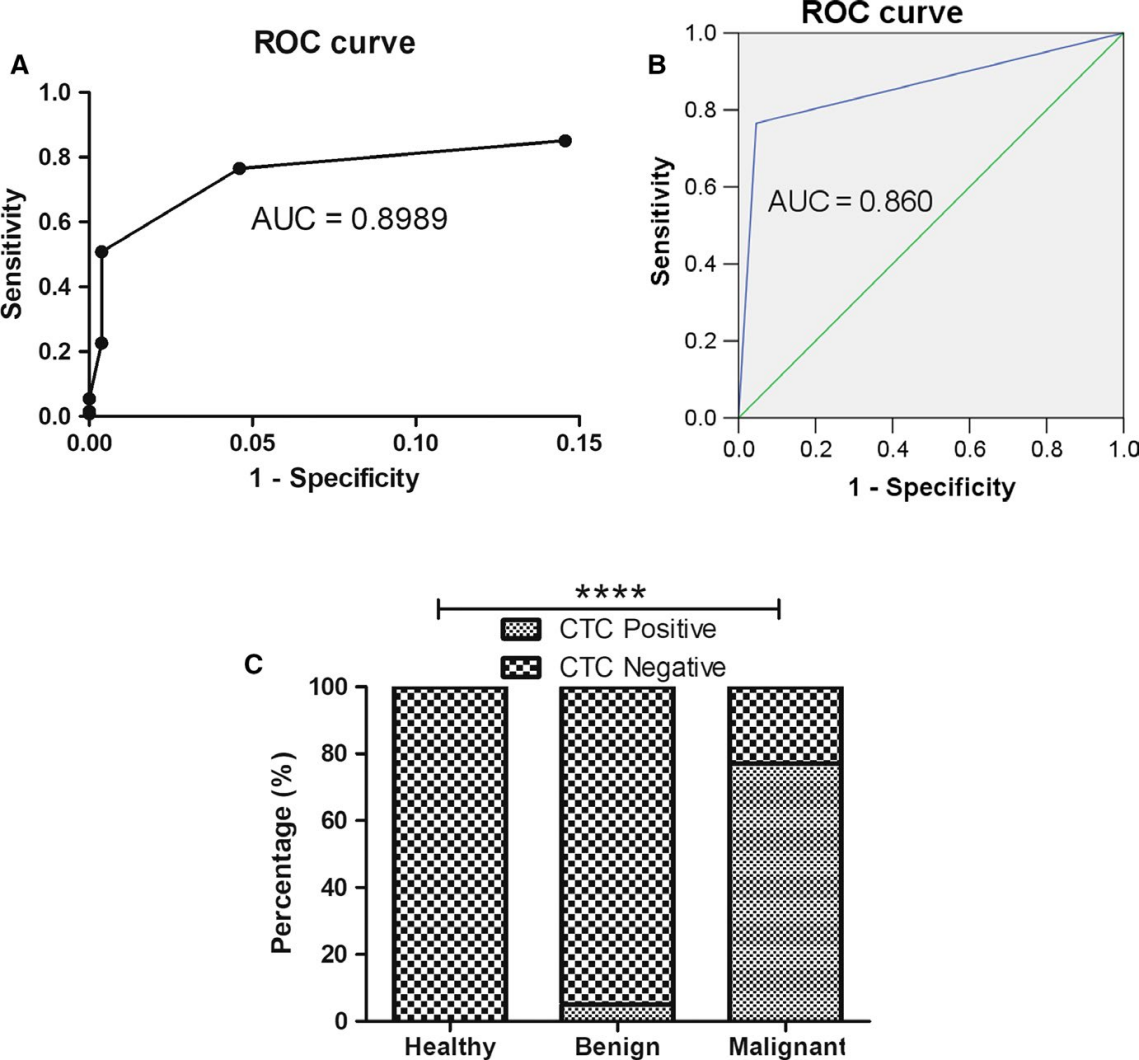
The diagnostic potency of CytoSorter^®^ circulating tumor cells (CTCs) detection system in breast cancer (BC) patients. A, The receiver operating characteristic (ROC) curve was plotted to evaluate the sensitivity, specificity, and area under the curve (AUC) of CytoSorter^®^ system, and to determine the CTC cut‐off value. The ROC curve gave an AUC of 0.8989 (*P* < .001). When CTC cut‐off value was set to 2, it gave the highest Youden index of 0.7196. B, When CTC cut‐off value was set to 2, the ROC curve gave an AUC of 0.86 with a specificity and sensitivity of 95.4% and 76.56%, respectively. C, When CTC cut‐off value was set to 2, the CTC‐positive rate can be used to distinguish BC patients from patients with benign breast disease and healthy volunteers (*P* < .0001). ****P < .0001

The ROC curve was plotted to evaluate the sensitivity, specificity, and AUC value of CytoSorter^®^ system, and the CTC cut‐off value was determined by Youden index. As shown in Figure 3A and Table 2, a CTC cut‐off value of 2 generated the highest Youden index of 0.7196. When CTC cut‐off value was set to 2, the ROC curve gave an AUC of 0.86 with a specificity and sensitivity of 0.954 and 0.7656, respectively. In order to assess whether CTC‐positive rate was associated with patients’ clinicopathological characteristic, Chi‐square analysis was performed among aforementioned different groups of patients, and results are shown in Table 3. Circulating tumor cell‐positive rate could be used to distinguish BC patients from the healthy volunteers and patients with BBD (*P* < .0001). However, there was no statistically significant correlation of CTC‐positive rate with age, cancer stage, TNM stage, cancer type, lymph node metastasis, or molecular subtypes.

**Table 2:** Youden index of different CTC Cut‐off values

CTC cut‐off (per 4 mL)	Sensitivity	Specificity	Youden index
1	0.8515	0.8544	0.7059
**2**	**0.7656**	**0.954**	**0.7196**
3	0.5078	0.9961	0.5039

Bold indicates cut off value. Abbreviation: CTC, circulating tumor cell.

**Table 3:** Statistical analysis of CTC‐positive rates among different groups of patients based on clinicopathological features

Subjects	N	CTCs ≥ 2	CTCs < 2	*x* ^2^	*P* value
Healthy	29	0	29	219.6	**<.0001**
Benign	232	12	220		
Malignant	128	98	30		
Cancer type					
Noninvasive	6	3	3	N/A	.1406
Invasive	122	95	27		
Cancer stage					
0	6	3	3	6.763	.149
I	46	35	11		
II	59	46	13		
III	16	14	2		
IV	1	0	1		
TNM stage					
Tumor stage					
Tis	6	3	3	3.903	.4193
T1	60	46	14		
T2	57	44	13		
T3	3	3	0		
T4	2	2	0		
Nodal stage					
N0	78	58	20	0.9091	.8232
N1	34	27	7		
N2	8	6	2		
N3	8	7	1		
Nodal metastasis					
No	78	58	20	N/A	.5257
Yes	50	40	10		
Molecular subtype^a^					
Luminal A	69	57	12	1.149	.7652
Luminal B	17	12	5		
HER2‐enriched	14	10	4		
Triple negative	21	16	5		

Bold indicates statistically significance values. Abbreviations: CTC, circulating tumor cell; N, number; N/A, not available; TNM, tumor node metastasis classification; *x* ^2^, chi‐square.

a Due to the missing information, seven patients were removed from this analysis.

## 4 DISCUSSION

Circulating tumor cells are considered to be a valuable prognostic predictor in BC.^9^, ^10^, ^11^ Circulating tumor cells can help monitoring patients' response to the treatment and tumor recurrences.^12^, ^13^ Studies have suggested that early diagnosis of tumor led to the improvement of survival of patients.^20^, ^21^ We aimed to investigate the feasibility of CTCs detection in BC patients using CytoSorter^®^ CTCs capture platform and to evaluate its clinical value in diagnosis of BC, especially regarding early diagnosis and cancer staging. A total of 366 patients suspected of having BC and 30 healthy volunteers were enrolled in this study, and CTC detection was performed before biopsy or treatment. Patients were later grouped into patients with BC and patients with BBD according to the biopsy results. Correlation of CTCs with patients' clinicopathological features was analyzed.

The results first indicated that CTCs could be used to distinguish BC patients from the healthy individuals and patients with BBD (*P* < .0001). CTCs are rare in healthy individuals or in patients with nonmalignant diseases.^22^ Circulating tumor cell studies in malignant head and neck cancer and pancreatic cancer using CytoSorter^®^ also shows that CTCs can be used to differentiate diseased patients from healthy people and patients with benign tumors.^18^, ^19^ More than 50 techniques have been developed to enrich CTCs in the peripheral blood, based on different physical parameters (size or density) and/or biological characteristics (cell surface markers) between CTCs and blood cells.^23^ Detection rates of CTCs in BC patients range from 8% to 55% depending on the detection method used.^24^ Schindlbeck et al used CellSearch^®^ to detect CTCs in 202 stage I‐IV BC patients, and the detection rate was 20%.^14^ Lucci et al used CellSearch^®^ as well to detect CTCs in 302 nonmetastatic BC patients, and the detection rate was 24%.^15^ Ma et al used flow cytometry to detect CTCs in 187 stage II‐III BC patients, and CTCs were identified in 80 patients (detection rate 42.78%).^25^ Daskalaki et al used reverse transcription polymerase chain reaction (RT‐PCR) method to detect CK‐19 mRNA‐positive CTCs in 165 stage I‐II BC patients, and CTCs were identified in 55.4% of patients.^26^ Molloy et al used a multimarker quantitative PCR‐based assay to detect CTCs in 733 stage I‐II BC patients, and CTC detection rate was only 7.4%.^27^ CTCs detection rate in BC with CytoSorter^®^ system in this study is 85.16%, which is much higher than any other previous studies. In early stage BC patients (cancer stage I and II), our CTCs detection rate can reach 80.43% and 91.53%, respectively, which is also much higher than other studies concerning CTC detection in early stage BC patients.^9^, ^24^ Furthermore, CTCs were detected in three of six BC patients with carcinoma in situ, the very early stage of tumor. Taken together, our results suggest that CytoSorter^®^ CTCs capture system has a greater sensitivity to detect CTCs in BC and it might be used as a biomarker to assist in the screening and early diagnosis of BC.

As CTCs could be used as a tool to distinguish patients with malignant tumors from patients with benign tumors as shown in Figure 1A, it implied that CTC enumeration should be able to reflect tumor burden. Comparing CTCs with BC patients' clinicopathological characteristics, we found that CTCs were correlated with cancer stage, tumor size, cancer type, and lymph node metastasis, but not with nodal stage. More CTCs were found in BC patients at advanced cancer stages and in BC patients with an invasive tumor, a bigger tumor, or lymph node metastasis. Our results are quite consistent with a pooled analysis of 3137 patients with nonmetastatic (stage I‐III) BC from five BC institutions, which used CellSearch^®^ system and showed that CTC‐positive patients had larger tumors, increased lymph node involvement, and a higher histologic tumor grade than CTC‐negative patients (all *P* < .002).^28^ Although there was no statistical significance between CTCs and nodal stages, we did observe more CTCs were found in patients with advanced nodal stage. The reason why there was no statistical significance might be due to that too few patients with N2 and N3 nodal stages were included in this study. We have only 1 stage IV BC patient in this study. Although CTC was successfully isolated in this patient, CTC count was 1, which is lower than the average CTC counts in stage I‐III patients. Studies have shown that CTCs undergoing epithelial mesenchymal transition would survive better in circulation and thus have a greater potential for metastasis.^29^ It is believed that patients with late stage tumors or metastatic tumors usually have more mesenchymal CTCs.^29^ Satelli et al have generated an Ab against cell‐surface vimentin (CSV) to detect specifically mesenchymal CTCs.^30^ They used EpCAM and CSV Abs separately to detect CTCs in MBC patients and found that CTC detection rate with CSV was higher than that with EpCAM, and CTC counts with CSV were more significant (*P* = .0053) in differentiating patients responsive and nonresponsive to treatment compared to CTC counts with EpCAM (*P* = .0564).^31^ In this study, EpCAM Ab was used to capture CTCs, and EpCAM Ab recognized only the epithelial type of cells. Therefore, the reason why less CTCs were detected in the stage IV BC patient might be that stage IV BC patients had mostly mesenchymal CTCs and they could not be captured by EpCAM Ab. CytoSorter^®^ system provides CSV mesenchymal CTCs capture kit as well. We should use this kit in BC to confirm whether CTC counts could reflect cancer and TNM stages. Although there was no significant correlation between CTCs and BC patients' molecular subtypes, the CTC detection rate with CytoSorter^®^ in each subtype was still higher than previously reported. Wang et al used RT‐PCR method to detect CTCs in 221 BC patients, and CTC detection rate in each molecular subtype were 35/55 (63.6%) for luminal‐A, 19/27 (70.4%) for luminal‐B, 41/56 (73.2%) for luminal‐B HER2‐positive, 13/17 (76.5%) for HER2‐positive (nonluminal), and 24/31 (77.4%) for triple‐negative.^32^ Circulating tumor cell detection in Stage I‐IV BC patients are, respectively, 80.43%, 91.53%, 87.5%, and 100%. And CTC counts are positively correlated with cancer stage. Taken together, CTCs could be used as a diagnostic aid to facilitate cancer staging.

Lastly, we liked to evaluate the diagnostic value of CytoSorter^®^ system in BC and to determine the CTC cut‐off value at which CytoSorter^®^ system has the greatest diagnostic potency. A CTC cut‐off value of 2 was found, which is consistent with the studies in pancreatic cancer 18 and the cancer screening project (unpublished data). Both studies showed that CTC cut‐off value of 2 could be used to differentiate diseased people from the healthy people and patients with benign diseases. When CTC cut‐off was set to 2, ROC curve gave an AUC of 0.86 with a specificity and sensitivity of 0.954 and 0.7656, respectively, whereas the specificity and sensitivity of CellSearch^®^ system in MBC patients were 0.8335 and 0.475, respectively,^31^ indicating that CytoSorter^®^ system has a better specificity and sensitivity than CellSearch^®^ system in BC. CTC‐positive rates analysis among different groups of patients based on their clinicopathological features showed there was no significant correlation of CTC‐positive rates with age, cancer stage, TNM stage, cancer type, or lymph node metastasis. It might be due to the high CTC‐positive rates in most of the groups and some statistical bias caused by the small sample size of certain group of patients.

Results of this study showed that CytoSorter^®^ system can successfully isolate CTCs in BC patients with a better sensitivity and specificity, and CTCs can be used as a tool to assist in cancer screening and early diagnosis. Studies have shown CTCs could be used as a prognostic factor and a monitoring aid for recurrence in BC as well.^9^, ^10^, ^11^, ^12^, ^13^ Thus, more studies on larger patient population with follow‐up should be conducted to elucidate the clinical value of CTCs as a diagnostic, therapeutic, and prognostic indicator in BC.

## Data Availability

I confirm that my article contains a Data Availability Statement even if no data is available (list of sample statements) unless my article type does not require one. I confirm that I have included a citation for available data in my references section, unless my article type is exempt.
